# Reactivity of NK Cells Against Ovarian Cancer Cells Is Maintained in the Presence of Calcium Phosphate Nanoparticles

**DOI:** 10.3389/fimmu.2022.830938

**Published:** 2022-02-18

**Authors:** Antonio Hrvat, Mathias Schmidt, Martin Obholzer, Sonja Benders, Sebastian Kollenda, Peter A. Horn, Matthias Epple, Sven Brandau, Nina Mallmann-Gottschalk

**Affiliations:** ^1^ Experimental and Translational Research, Department of Otorhinolaryngology, University Hospital Essen, Essen, Germany; ^2^ Inorganic Chemistry and Center for Nanointegration Duisburg-Essen (CeNIDE), University of Duisburg-Essen, Essen, Germany; ^3^ Institute for Transfusion Medicine, University Hospital Essen, Essen, Germany; ^4^ German Cancer Consortium, Partner Site Essen-Düsseldorf, Essen, Germany; ^5^ Department of Gynecology and Obstetrics, University Hospital Essen, Essen, Germany

**Keywords:** NK cells, nanoparticles, ADCC, calcium phosphate, cetuximab, aggregation, ovarian cancer, immunotherapy

## Abstract

Calcium phosphate nanoparticles (CaP-NPs) are biodegradable carriers that can be functionalized with biologically active molecules. As such, they are potential candidates for delivery of therapeutic molecules in cancer therapies. In this context, it is important to explore whether CaP-NPs impair the natural or therapy-induced immune cell activity against cancer cells. Therefore, in this study, we have investigated the effects of different CaP-NPs on the anti-tumor activity of natural killer (NK) cells using different ovarian cancer (OC) cell line models. We explored these interactions in coculture systems consisting of NK cells, OC cells, CaP-NPs, and therapeutic Cetuximab antibodies (anti-EGFR, ADCC-inducing antibody). Our experiments revealed that aggregated CaP-NPs can serve as artificial targets, which activate NK cell degranulation and impair ADCC directed against tumor targets. However, when CaP-NPs were properly dissolved by sonication, they did not cause substantial activation. CaP-NPs with SiO2-SH-shell induced some activation of NK cells that was not observed with polyethyleneimine-coated CaP-NPs. Addition of CaP-NPs to NK killing assays did not impair conjugation of NK with OC and subsequent tumor cytolytic NK degranulation. Therapeutic antibody coupled to functionalized CaP-NPs maintained substantial levels of antibody-dependent cellular cytotoxic activity. Our study provides a cell biological basis for the application of functionalized CaP-NPs in immunologic anti-cancer therapies.

## Introduction

Nanomedicine has developed into an emerging field of research originating from applied biomedicine and novel nanotechnology (1). Due to their specific physical and biochemical properties nanoparticles offer new opportunities for diagnostic and therapeutic applications, e.g. in cancer therapy or autoimmune diseases (2, 3).

Besides commonly used inorganic nanoparticles like gold or iron oxide nanoparticles, calcium phosphate nanoparticles (CaP-NP) are also suitable for application in the medical field. As calcium phosphate is an endogenous biomineral with natural presence in human tissue such as bones and teeth, CaP-NPs display high biocompatibility and low intrinsic toxicity (4). CaP-NPs are clinically applied in traumatology, endoprosthetic applications, and tissue engineering: They can support the regeneration of bone defects and mediate bone contact in biomedical implants (4) (5). Ca-NPs have also been incorporated in different polymers to enhance the biomechanical properties of scaffolds for hard tissue regeneration (6). Additionally, they are valuable components in toothpastes supporting tooth repair and remineralization (7).

CaP-NPs show high chemical stability, especially at physiological pH in tissue or blood. Therefore, in the last decades, CaP-NP have been developed to be also utilized as vehicles for different cargo molecules. They served as carriers for antibiotics and have been applied for vaccination and immunization against infectious diseases successfully (8). In contrast to other nanoparticle-preparations, CaP-NP can be loaded with various immune activating components at flexible dosages simultaneously, which optimizes and individualizes the immune response (9). Taken up by dendritic cells CaP-NPs were able to induce a strong immunization *in vivo* and showed efficiency against experimental retroviral infections (10).

CaP-NPs are also promising tools for gene therapy which predestined them to become part of immunotherapeutic concepts in cancer therapy. To this end, DNA and short interfering RNA (siRNA) have been incorporated in CaP-NP for transfection in eukaryotic cells successfully as nucleic acids are otherwise unable to cross the cell membrane and are rapidly degraded by specific nucleases (11). Compared to other well-established transfection agents such as Lipofectamine CaP-NP displayed comparable transfection efficacy but significantly less cytotoxicity which is beneficial for application *in vivo* (12). In pancreatic cancer, VEGF-siRNA-loaded CaP-NP induced high gene silencing efficiency without associated toxicity with consecutive reduction of tumor growth (13). PEGylated siRNA-loaded CaP-NP containing also doxorubicin resulted in enhanced cell apoptosis and tumor growth arrest *in vivo* (14). The inclusion of dyes or imaging components in CaP-NP facilitates the visualization of the tumor tissue. For example, a MRI contrast agent encapsulated into CaP-NP enhanced the sensitivity of detection of primary hepatocellular carcinoma compared to a standard contrast agent (15). Additionally, the covalent binding of specific antibodies on the surface of CaP-NP enables functionalization of the particles and offers the possibility for individualized targeted cancer therapy (8).

For the therapeutic purpose, it is fundamental that nanocarriers in the physiological environment do not impair the activity of immune cells or diminish the efficacy of their incorporated agents. However, the mechanisms of interactions between nanoparticles and physiological components are still poorly understood (16) (17). According to nanotoxicological studies, CaP-NPs unspecifically adsorb proteins to the surface forming “protein-corona” due to the high protein concentration physiological environment *in vivo* (18). Further studies could show that CaP-NPs may agglomerate depending on size, charge and surface characteristics which resulted in altered biodistribution, cellular uptake, and toxicity of CaP-NPs in blood and tissue (8).

Despite obvious relevance for therapeutic applications, interactions between CaP-NPs and different immune effector cells have not been studied intensively in the past. Many studies focused on interactions between CaP-NPs and macrophages, and little, if any, data are available on the interplay of Ca-P NPs with T cells or NK cells (9). As part of the innate immune system, NK cells are likely to be one of the first cells coming into contact with nanoparticles when those are systemically applied. Physiologically, NK cells exert direct, natural cytotoxicity to target cells that are altered by infection or malignant transformation. Here, loss of MHC class I-molecules or upregulation of stress-induced ligands such as MICA/B (MHC class I polypeptide-related sequence A/B) and UL16 binding proteins 1—6 (ULBP 1—6) on target cells are crucial triggers inducing NK cell-activation and -cytotoxicity. Thereby, NKG2D and DNAM-1 as well as NKp46, NKp44 and NKp30 are central corresponding receptors on NK cells, whereas CD69 reflects their general activity state (19). By recognizing and binding antibody-coated cells *via* CD16, NK mediate antibody-dependent cellular cytotoxicity (ADCC) and thus achieve the maximum cytotoxic activity. Finally, they lyse target cells *via* inducing apoptosis or releasing perforin/granzymes as reflected by the expression of the lysosomal-associated membrane protein-1 LAMP-1 or CD107a on the cell surface (20, 21). NK cells support their activity and regulate other immune cell responses by the secretion of various cytokines like IFNγ or TNFα. Because of these comprehensive antitumoral properties, NK cells represent important cellular components in many immunotherapeutic approaches.

In this study, we analyzed whether CaP-NPs affect natural and antibody-dependent cytotoxicity of NK cells against ovarian cancer cells. To this end, we studied the effects of CaP-NP on interactions between NK cells and tumor cells as well as the direct effects on NK and tumor cells. For our experiments, we used previously established protocols for CaP-NP-preparation including triple-shell CaP-NPs as well as silica shell-coated CaP-NPs (22). For visualization, we utilized fluorescent CaP-NPs and tested also functionalized CaP-NPs. These were bound to the antibody Cetuximab which is directed against EGFR (epidermal growth factor receptor), a common target in ovarian cancer cells.

## Materials and Methods

### Synthesis and Functionalization of Calcium Phosphate Nanoparticles

Single-shell CaP-NPs (CaP) were synthesized by precipitation using 0.25 mL of 6.25 mM calcium nitrate solution (Ca(NO3)2·4 H2O) and 0.25 mL of 3.74 mM diammonium hydrogen phosphate ((NH4)2HPO4) solution (both solutions were adjusted to pH 9.0 with NH3) and 0.05 mL of labelled poly(ethyleneimine) solution diluted with non-labelled PEI 1:5 (PEIFITC/Cy5: 2 mg mL-1, MW 25 kDa, branched, Surflay) or 0.05 mL non-labelled poly(ethyleneimine) solution (PEI: 2 mg mL-1, MW 25 kDa; branched, Sigma-Aldrich) (22). For the synthesis of triple-shell particles (CaP-P) the same procedure of calcium phosphate precipitation and PEI coating was repeated with the single-shell particles dispersion. These generated nanoparticles were centrifuged for 15 min at 12,000 g (MiniSpin^®^, Eppendorf). Then, the particle pellet was redispersed in 1 mL UltraPureTM DNase/RNase-free water in an ultrasonic water bath (Elmasonic S10, Elma) for 10-15 s.

Silica-shell CaP-NPs (CaP-S) were synthesized for a sequential surface functionalization with antibodies. The synthesis was performed according to previously reported procedure (23). Initially, 20 mL of absolute ethanol were mixed with 0.013 mL of a 30% aqueous ammonium solution and 0.025 mL of Tetraethyl orthosilicate (TEOS) and stirred for 10 min. Next, 5 mL of a dispersion of single-shell CaP-NPs (either stabilized with fluorescently labelled or non-labelled PEI) were pipetted into the ethanol mixture and stirred overnight at room temperature. The crude dispersion of silica-shelled CaP-NPs was centrifuged for 30 min at 66,000 g and redispersed in 5 mL UltraPureTM DNase/RNase-free water by ultrasonication for 10-15 s. Then, the redispersed nanoparticles were added to 20 mL of absolute ethanol containing 0.025 mL of 3-mercaptopropyl) trimethoxysilane (MPS), for a subsequent surface functionalization, and stirred for 6 h at room temperature. The thiol-terminated nanoparticles (CaP-S) were centrifuged and redispersed as described previously. For 1 mL of CaP-S dispersion 500 µg of the Cetuximab (Merck) were coupled to the particle surface overnight. Therefore, the antibodies were incubated in 1 mL of PBS containing 0.025 mL of a 20 mM N Succinimidyl-3 (2-pyridyldithio) propionate (SPDP) solution for 1 h. The reaction mixture was desalted using centrifugal filters with a molecular weight cutoff of 100 kDa (Amicon^®^ Ultra – 0.5 mL, Merck). The activated antibodies were pipetted into the dispersion of CaP-S for the conjugation of the thiol-reactive part of SPDP to the thiol groups on the nanoparticles, incubated overnight at room temperature and the next day centrifuged for 10 min at 12,000 g. The supernatant of these nanoparticles (CaP S C) was used to calculate the number of coupled antibodies per particle. For the long-time storage of all synthesized nanoparticles the final dispersions of each species were supplied with 20 mg mL-1 D-(+)-trehalose (Sigma Aldrich), which serves as a cryoprotectant, and aliquoted to 0.1 mL, shock-frozen with liquid nitrogen and freeze dried with an Alpha- 2-4 LSC system (Christ).

### Characterization of Calcium Phosphate Nanoparticles

The number of particle-coupled antibodies was determined with a DS 11 FX+ Nanodrop instrument by UV/Vis spectroscopy of the supernatant (uncoupled antibodies in the supernatant were subtracted from the applied mass). The morphology and size of the solid core diameter of the synthesized nanoparticle species were characterized by scanning electron microscopy (ESEM Quanta 400, FEI and Apreo S LoVac, Thermo Fisher Scientific) after palladium-gold sputtering. Their hydrodynamic diameter and zeta potential were determined by dynamic light scattering (Zetasizer Ultra, λ=532 nm backscatter mode, Malvern Panalytical). All displayed particle size data refer to scattering number distributions. To calculate the number of particles per milliliter the calcium concentrations of the nanoparticles were determined by atomic absorption spectroscopy (AAS; M-Series AA spectrometer; Thermo Electron Corporation) after dissolution of the samples in hydrochloric acid. The calculated nanoparticle concentration was combined with the total amount of coupled antibodies, if applicable, and brought into perspective.

### Ovarian Cancer Cell Lines and *In Vitro* Cell Culture

SKOV-3 and OVCAR-3 ovarian cancer cell lines were kindly provided by the Department of Obstetrics and Gynecology, University of Bonn, Germany. OVCAR-4 was obtained from Westdeutsches Tumorzentrum, University of Duisburg-Essen, Germany. SKOV3 ovarian cancer cell line was cultured in Roswell Park Memorial Institute (RPMI Gibco). OVCAR4 ovarian cancer cell line was cultured in a mixture of ¾ of RPMI and ¼ of Dulbecco’s Modified Eagle Medium (DMEM). Both media were supplemented with 10% (v/v) heat-inactivated fetal calf serum (FCS Gibco), 100 U/mL penicillin, and 100 mg/mL streptomycin (PenStrep, Gibco by Life Technologies) (supplemented complete media). Cells were cultivated in plastic flask (Sarstedt) at 37°C and 5% CO2 and continuously passaged by treatment with Accutase (Gibco) for 5 minutes at 37°C.

### NK Isolation From Peripheral Blood

Healthy donor blood collected in trisodium citrate blood collection tubes was diluted with Dulbecco’s Phosphate Buffered Saline (DPBS, Gibco, Life Technologies Limited) in 1:1 ratio and overlayed on a 1.077g/mL separation medium (Biocoll, Merck Millipore). Density centrifugation was performed at room temperature (400g for 30min) without acceleration and brake. PBMCs were collected and washed with PBS followed by centrifugation at 300g for 8min at room temperature. Then plastic adherence was performed to deplete monocytes by incubating them in a T175 flask (Sarstedt) at 37°C and 5% CO2 for 1 hour. For NK isolation NK MACS Isolation Kit (Miltenyi Biotec) was used according to the manufacturer’s instructions. Purity of isolated NK cells was routinely tested and ranged from 90% to 97%. After isolation 5 U/ml recombinant human IL-15 (50µg, Immuno Tools) was added to NK cells that were used for functional experiments after overnight incubation.

Use of peripheral blood from healthy donors was approved by the institutional review board of the Medical Faculty of the University of Duisburg-Essen (approval number 07-3500 and 08-3590) and each donor signed an informed consent form.

### NK Degranulation Assay

NK cells express CD107a during degranulation, which also correlates to NK cell-mediated tumor cell lysis (21). To evaluate natural and antibody-dependent NK cell cytotoxicity purified NK cells and SKOV-3/OVCAR-4 cells were coincubated (1:1 ratio) in a flat-bottom 96-well plate with or without different nanoparticles. For ADCC-experiments Cetuximab 1 μg/ml (Erbitux, 5mg/mL, Merck (Serono)) or Cetuximab-bound nanoparticles in corresponding concentrations were added. NK cells were labelled with anti-CD107a-FITC (25µg/mL, clone H4A3, Mouse IgG1, k, BD Biosciences). After incubation for 1 hour at 37°C and 5% CO2, the protein Golgistop-Monesin (BD Biosciences) was added (1:600). After further 5 hours incubation NK cells were stained with CD56-BV421 (12µg/mL, NCAM 16.2, IgG2b,k, BD Biosciences) and CD107 expression analyzed by flow cytometry.

NK cell degranulation was calculated by the following formula in case of natural cytotoxicity:


NC (without NPs)=(NK+TC)−NK

NC (with NPs)=((NK+TC+NP)−NK)−NP

NP or NP induced degranulation was calculated as:


NP=(NK+NP)−NK.

For antibody-dependent cell-mediated cytotoxicity the following formula was used:


ADCC (without NPs)=(NK+TC+CET)−NK−NC (without NPs)

ADCC (with NPs)=(NK+TC+CET+NP)−NK−NP−NC (with NPs)

### Nanoparticle Cytotoxicity and Tumor-Killing Assay

NK cells and SKOV-3/OVCAR-4-cells were coincubated (1:1 ratio) with or without Cetuximab (1 µg/ml) and different types of nanoparticles for 24h at 37°C and 5% CO2. Adherent and suspended cells were harvested with Stem Pro Accutase (Gibco by Life Technologies). After washing step cells were stained using PE Annexin V Apoptosis Detection Kit I (BD Biosciences) according to the manufacturer’s protocol and analyzed by flow cytometry. Alive cells were defined as Annexin V-/7AAD-. Tumor cell lysis was calculated by the following formula in case of natural cytotoxicity:


NC (without NPs)=(TC+NK)−TC

NC (with NPs)=(TC+NK+NP)−TC−NP

NP or NP induced killing was calculated as:


NP=(TC+NP)−TC

For antibody-dependent cell-mediated cytotoxicity the following formula was used:


ADCC (without NPs)=(TC+NK+CET)−TC−NC (without NPs)

ADCC (with NPs)=(TC+NK+CET+NP)−TC−NP−NC (with NPs)

### Flow Cytometric Analysis of NK Cell and Tumor Cell Markers

After six hour coincubation with nanoparticles, the following antibodies for the flow cytometric analysis of the NK cell marker expression were used: CD56-BV421 (12µg/mL, clone NCAM 16.2, mIgG2b,k, BD Biosciences), CD16-BV510 (180µg/ml, clone 3G8, mIgG1, Biolegend), NKp46-PE (CD335, 50µg/ml, clone 9E2, mIgG1, k, Biolegend), DNAM-1-PerCP-Cy5.5 (CD226, 200µg/ml, clone 11A8, mIgG1, k, Biolegend), NKG2D-PE-Cy7 (CD314, 200µg/ml, clone 1D11, mIgG1, k, Biolegend), CD69-FITC (100µg/ml, clone FN50, mIgG1, k, Biolegend), followed by a live/dead staining using the fixable viability dye eFluor 780 (eBioscience/Thermo Fisher Scientific, Darmstadt, Germany). For intracellular staining with anti-IFNγ-APC (7.5µg/ml, clone 45-15, mIgG1, k, Miltenyi), cells were fixed and permeabilized with BD Cytofix/Cytoperm Solution Kit (BD Biosciences). Before NK stimulation, Golgistop-Monesin (BD Biosciences) was added to the cells. Ovarian cancer cell surface marker expression were detected by staining with the following antibodies: MICA-APC (5µg/ml, clone 159227, mIgG2b, k, RD Systems), UBLP-2/5/6-APC (10µg/ml, clone 165903, mIgG2a, k, RD Systems), CD54-PE (100µg/ml, clone HA58, mIgG1, k, Biolegend), and MHCI-PE (25µg/ml, clone W6/32, mIgG2a, k, Biolegend). The same fixable viability dye as for NK markers was used. In all flow cytometry measurements appropriate isotype controls were used: mIgG1-BV510 (100µg/ml, clone MOPC-21, Biolegend), mIgG1-PE (50µg/ml, clone MOPC-21, BD Bioscience), mIgG1-PE-Cy-7 (200µg/ml, MOPC-21, Biolegend), mIgG1-FITC (500µg/ml, clone MOPC-21, Biolegend), mIgG1-PerCP-Cy5.5 (200µg/ml, clone MOPC-21, Biolegend), mIgG1-APC (200µg/ml, clone MOPC-21, Biolegend), mIgG2b-APC (200µg/ml, clone MPC-11, Biolegend), mIgG2a-APC (200µg/ml, clone MOPC-173, Biolegend), mIgG2a-PE (200µg/ml, clone MOPC-173, Biolegend). Stained cells were analyzed with BD FACSCanto II using DIVA 8.01 software (BD Biosciences) or FlowJo10 (LLC, Ashland, Oregon, USA).

### Detection of NK IFNγ -Secretion by ELISpot

The ELISpot-technique was applied for sensitive detection of IFNγ-secreting NK cells. First, Multiscreen 96-well filtration plate (Merck Millipore) was activated with 35% ethanol and coated with anti-IFNγ-capture antibody (200µg/ml, clone 1-D1K, mIgG1, k, Mabtech). After incubation at 4°C overnight the plates were blocked with 200µl of supplemented complete RPMI-1640-media for 2h at 37°C. After washing step isolated NK cells were seeded in triplicates and treated with different nanoparticle types. NK cells treated with PMA (50ng/ml, Sigma-Aldrich) and Ionomycin (1µg/ml, Sigma-Aldrich) were included for positive control. Untreated NK cells were used as negative control. After incubation with 12,5 µl of calcium phosphate nanoparticles for 24 h at 37°C and 5% CO2 plates were washed in the ELISA-Washer (PBS/0,05% Tween-20.) Biotinylated anti-IFNγ-detection antibody (200µg/ml, clone 7-B6-1, mIgG1, k, Mabtech) was added in 2µg/ml PBS and 1% BSA. The plates were incubated for 2 h at 37°C, washed and incubated with 50µl ExtraAvidin alkaline phosphatase (1:1000 diluted in PBS/1% BSA, Sigma-Aldrich) for 2h at the room temperature. After washing steps 75µl of the ELISpot substrate BCIP/NBT (Roche) was added and incubated for 5-10 minutes. Developed cytokine spots were measured using AID Classic ELISpot Reader and the results were analyzed with AID ELISpot 7.0 software.

### ELISA Analysis of Tumor Cell-Secreted Cytokines

The supernatant of 50.000 OVCAR4 and SKOV3 cancer cells incubated with 12,5 µl of calcium phosphate nanoparticles for 24 hours was used to quantify secreted IL-6 and IL-8 using ELISA kits (R&D Systems, Wiesbaden, Germany), according to the manufacturer’s instructions.

### Conjugation Assay

Adapted from existing literature (24), the conjugation rate of NK and tumor cells was analyzed in presence of nanoparticles. NK cells were stained with Cell Tracker Red (Thermo Fisher Scientific) and tumor cells with Cell Tracker Green (Thermo Fisher Scientific) according to the manufacturer’s instructions. NKs (50.000 cells) and tumor cells (200.000 cells) were coincubated (1:4 ratio) in presence of 1 μg/ml Cetuximab with or without different types of CaP-NPs in matching concentrations. After centrifugation (20g (570 rpm), 1 min) tubes were placed in a water bath at 37°C for 45 min. Afterward, the samples were retrieved and briefly vortexed. 300 µl of ice-cold 0,5% PFA in PBS was added and samples were analyzed by flow cytometry.

### Live-Cell Microscopy of the NK-Tumor Cell-Calcium Phosphate Nanoparticle Interaction

For microscopy, tumor cells, seeded on 4 Chamber slides (LabTek) the day before, were stained with 500 µl of a 250 nM Calcein-AM (BD Pharmingen) solution in PBS 3% HS for 30 min in a darkened incubator. After washing, tumor cells were incubated in medium without phenol red in the dark. NK Cells were added in a 1:1 ratio and treated with 50 μl of fluorescent Cy5-CaP-NPs. For Live cell imaging, the Zeiss AxioObserver.Z1 at the Imaging Center Essen (IMECS) was used. Pictures of the pre-warmed chamber (37°C for 30 min) were taken every 4 minutes up to 4 hours. Representative position of each well and pictures with FITC, Cy5, and transmitted light channels were recorded. Digital processing was performed with ImageJ.

### Microscopy of the Calcium Phosphate Uptake by Tumor Cells

SKOV3 and OVCAR4 cells were seeded on coverslips and incubated in media at 37°C and 5% CO2 until confluent, roughly 72 hours. After replacing media 50µl of FITC-labeled CaP-S and CaP-S-C were added and incubated for 6 hours. Cells were washed and resuspended in PBS supplemented with 3% of human serum. Fixation was done in Cytofix/Cytoperm (BD-Bioscience) at room temperature in the dark for 15 min. After washing steps with Permwash (BD-Bioscience) cells were stained with 1:36000 DAPI/Permwash staining solution at room temperature for 10 min in the dark followed by washing with PBS. Stained coverslips were transferred on top of a drop of mounting liquid (VECTA) cells facing down, stored at 4°C in a sealed box overnight and scanned with AxioScan (Zeiss) at 488 nm (FITC) and 405 nm (DAPI).

### Flow Cytometry Analysis of Calcium Phosphate Nanoparticle Uptake in Tumor and NK Cells

For analyzing the uptake of nanoparticles in SKOV3/OVCAR4, the tumor cells were seeded the day before and incubated with 8, 12 or 20 μl of FITC labeled CaP-S or CaP-P and Cap-S-C in matched particles number, respectively. Incubation was performed in the dark, at 37°C, 5% CO2 for 6h. For analyzing preferential uptake coculture of SKOV3 and NK cells was incubated with 12,5 μl of different FITC-labeled CaP-NPs (CaP-P, CaP-S and CaP-S-C) for 3h at 37°C and 5%CO2. After washing step adherent cells were harvested using Accutase (Gibco) and resuspended with suspended cells in PBS 3% HS for analysis in the flow cytometer. FITC-positive cells were defined as cells that bind or take up CaP-NPs.

### Statistical Analysis

**Figure 1:**
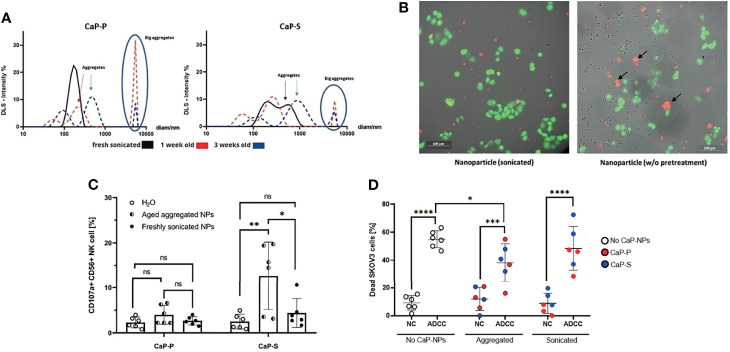
Non-sonicated aggregated CaP-NPs serve as artificial targets that activate NK cells. **(A)** Size and particle dispersion of sonicated and agglomerated CaP-NPs determined by dynamic light scattering. **(B)** OC cells, NK cells and CaP-NPs were co-incubated for 3 h and subjected to fluorescence microscopy. OC cell line – calcein green, NK cells – no fluorescence, CaP-NPs – Cy5-red. 20x. Left panel – sonicated; Right panel – not sonicated. NP aggregates shown by black arrows. **(C)** NK cells were incubated with 12,5 μl of sonicated or aggregated CaP-P and CaP-S nanoparticle suspensions for 6 h and NK cell degranulation was determined by CD107a surface staining. Every data point represents a different healthy NK cell donor, N=8 donors. **(D)** SKOV3 OC cells were coincubated with NK cells (NC), Cetuximab (ADCC), and sonicated or aggregated CaP-P-NPs or CaP-S NPs for 24 hours and OC cell death was determined by Annexin/7AAD staining. Every data point represents a different healthy NK cell donor, N=3. Significance testing was done using ordinary one-way ANOVA and posthoc Dunnett’s multiple comparisons test, significance is assumed for p < 0,05 (*), <0,01 (**), <0,001 (***), <0,0001 (****). No significance between compared groups indicated by ns.

**Figure 2:**
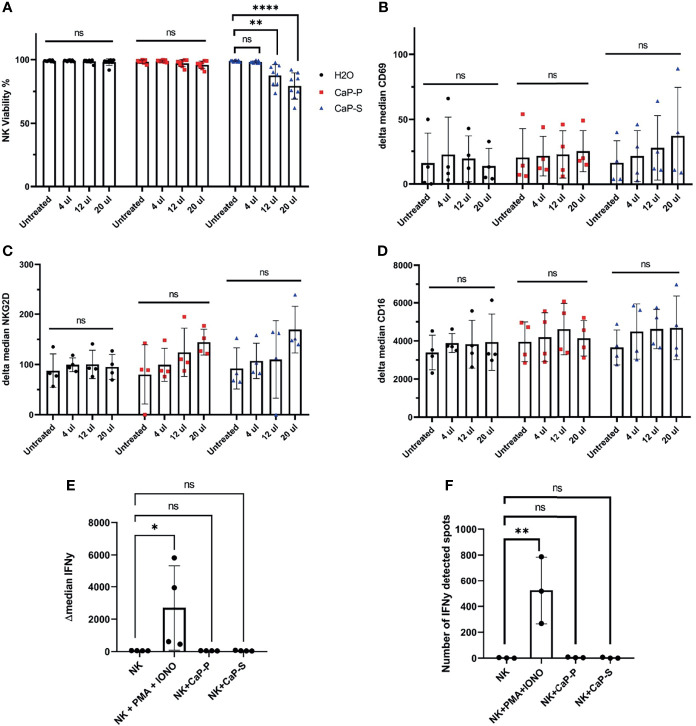
Sonicated and dispersed CaP-NPs do not induce NK cell activation. NK cells were incubated for 6 hours with different volumes of sonicated CaP-NPs or water as vehicle control and **(A)** NK cell viability was determined by Annexin/7AAD staining and flow cytometry. Data points represent four different NK donors and two technical replications, N=8. After 6h stimulation with CaP-NPs changes in expression of NK cells activation marker CD69 **(B)**, NKG2D **(C)**, and Fcγ receptor CD16 **(D)** were determined by surface staining flow cytometry. Data points represent four different NK cell donors, N=4. After stimulating NK cells with 12.5µl CaP-P or CaP-S sonicated nanoparticles for 6 hours **(E)** IFNγ production was determined by intracellular flow cytometry staining. Data points represent 4 different NK cell donors, N=4. **(F)** NK cell secretion of IFNγ after stimulation with 12.5 μl of CaP-NPs for 24 hours as determined by ELISpot. Y-axis indicated the number of cells or spots positive for IFNγ presence. Data points represent 3 different NK cell donors, N=3. As a positive control in both experiments, NK cells treated with PMA (50ng/ml) and Ionomycin (1µg/ml) were included. Significance testing was done using ordinary one-way ANOVA and posthoc Dunnett’s multiple comparisons test, significance is assumed for p < 0,05 (*), <0,01 (**), <0,0001 (****). No significance between compared groups indicated by ns.

**Figure 3:**
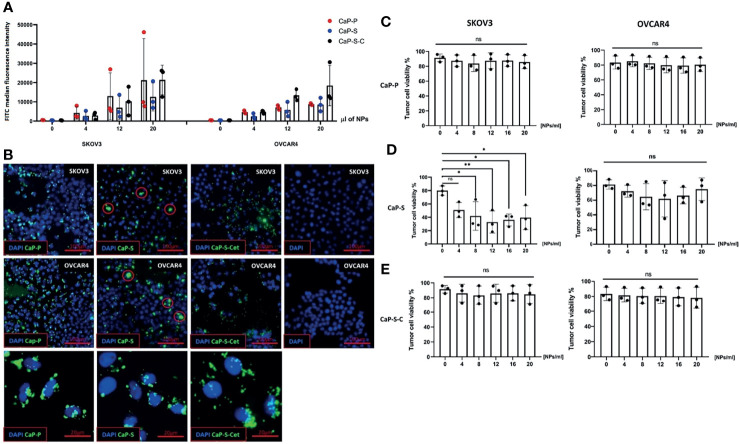
Internalization of CaP-NPs by ovarian cancer cells and cytotoxicity. **(A)** Ovarian cancer cells were exposed to different amounts of FITC labeled CaP-NPs and nanoparticle uptake was measured by flow cytometry after 6 h post addition. Y-axis shows FITC median fluorescence intensity. **(B)** Representative fluorescent microscopy images of different CaP-NP (green FITC label) uptake 3h after addition to ovarian cancer culture (top row - SKOV3, middle row - OVCAR4, bottom row - magnified details from OVCAR4 images. CaP-S aggregates marked with red circle. 40x magnification. Ovarian cancer cell lines (OVCAR4 and SKOV3) were incubated for 24 h with the different volumes of **(C)** CaP-P **(D)** CaP-S and **(E)** CaP-S-C nanoparticles and cell viability was determined by Annexin/7AAD staining. Each data point represents individual experiments, N=3. Statistical analysis was done using ordinary one-way ANOVA and posthoc Dunnett’s multiple comparisons test, significance is assumed for p < 0,05 (*), <0,01 (**). No significance between compared groups indicated by ns.

**Figure 4:**
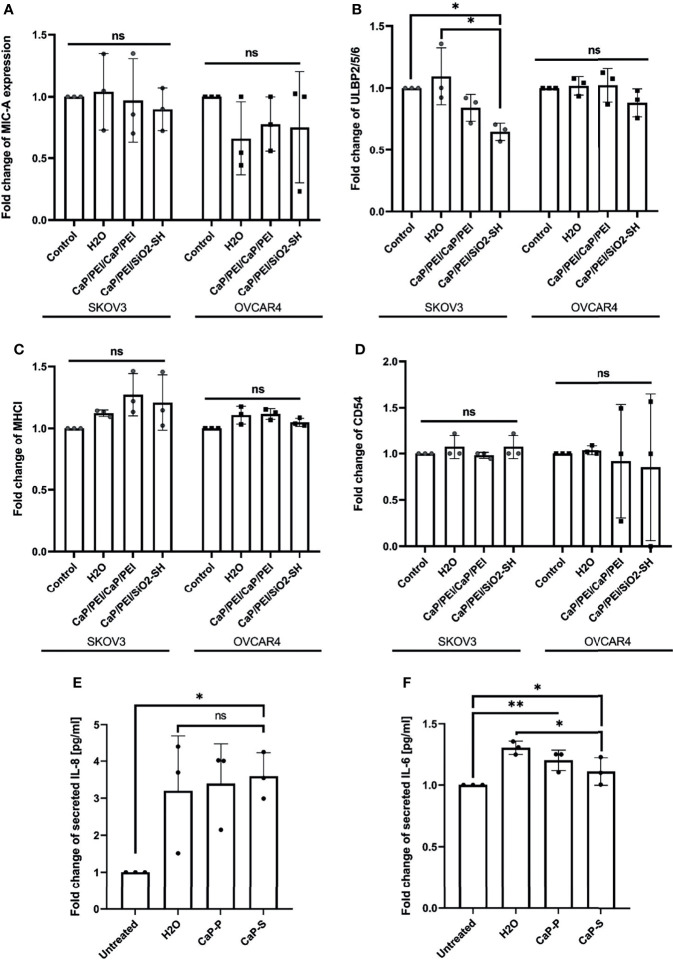
CaP-NPs do not affect ovarian cancer cell stress ligands and immune related markers. Ovarian cancer lines (OVCAR4 and SKOV3) were coincubated with 12.5 μl of sonicated CaP-P or CaP-S suspensions for 24 hours after which cell culture supernatant was collected and expression of stress ligands **(A)** MICA/B, **(B)** ULBP-2,5,6 and expression of **(C)** MHCI and **(D)** CD54 were determined by flow cytometry. Y-axis shows delta median. Each data point represents individual experiments, N=3. The amount of proinflammatory cytokines **(E)** IL-8 and **(F)** IL-6 secreted from SKOV3 (OVCAR4 secretion was below detection) was determined by ELISA. Each data point in the figure represents individual experiments, N=3. Significance testing was done ordinary one-way ANOVA test with posthoc Dunnett’s multiple comparison tests, significance is assumed for p < 0,05 (*), <0,01 (**). No significance between compared groups indicated by ns.

**Figure 5:**
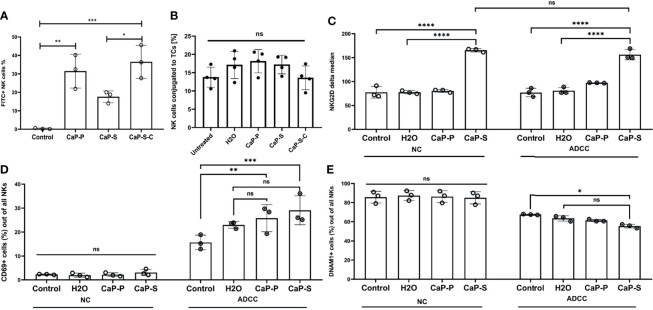
Presence of CaP-NPs does not interfere with the NK-target cell interaction. **(A)** NK-TC 1:1 coculture was coincubated with 12.5 µl of FITC labeled CaP-NPs for 3 h and percentage of NK cells that take up CaP-NPs was determined by flow cytometry. **(B)** NK cells and SKOV3 cells were mixed in a 4:1 effector to target ratio in the presence of 1 μg/ml soluble Cetuximab or a corresponding concentration of antibody that was coupled to NP (CaP-S-C). After 45 minutes of coincubation, the percentage of the conjugated NK cells was measured by flow cytometry. Each data point represents an individual NK cell donor, N=4. Significance testing was done using ordinary one-way ANOVA and posthoc Dunnett’s multiple comparisons test, significance is assumed for p < 0,05 (*), <0,01 (**), <0,001 (***). NK and TC were co-cultured in 1:1 ratio and stimulated with 12.5 µl of sonicated CaP-NPs for 6h. Expression of NK cell activation markers **(C)** NKG2D, **(D)** CD69, and **(E)** DNAM-1 was assessed by surface staining and flow cytometry. In all experiments, each data point represents NK cells from an individual healthy donor, N=3. Significance testing was done using ordinary two-way ANOVA and posthoc Sidaks multiple comparisons test, significance is assumed for p < 0,05 (*), <0,01 (**), <0,001 (***), <0,0001 (****). No significance between compared groups indicated by ns.

Data are shown as single values, means as center values and error bars for the standard deviation (SD). The ordinary one-way ANOVA test with posthoc Dunnets multiple comparison tests was used to statistically evaluate the difference between the groups (**Figures 1C, D**, **Figures 2A–F**, **Figures 3C, E**, **Figures 4A–G**, **Figures 5A, B**). A two-way ANOVA test with posthoc Sidaks multiple comparison tests were used to statistically evaluate the difference between more than two groups (**Figures 5C, E**). Significance testing was also done using both unpaired t-test with Welch’s correction and one-way ANOVA with posthoc Dunnets multiple comparison test (**Figures S3** and **S4**). Calculations were performed using GraphPad Prism 8 software.

## Results

### Calcium Phosphate Nanoparticles Are Biocompatible State of the Art Produced Nanoparticles

Multi-shell nanoparticles were prepared by precipitation of calcium phosphate (**Figure S1A**). Subsequently, the nanoparticle surface was functionalized with PEI (CaP-P) (**Figure S1B**) and in some cases with silica (CaP-S) (**Figure S1C**). Functionalization with PEI was performed to enable incorporation of nucleic acids and fluorescent molecules. Silica functionalization enabled conjugation of cetuximab anti-EGFR-antibodies onto the nanoparticle surface (CaP-S-C) (**Figure S1D**). Average hydrodynamic particle diameters were determined by dynamic light scattering (DLS) to be 129 ± 32 nm for CaP-P, 309 ± 49 nm for CaP-S and 97 ± 19 nm for CaP-S-C (**Figure S1E**). Use of FITC labeled PEI did not change the hydrodynamic radius (**Table S1**). Zeta potential of CaP-NPs was between +18 and +28 mV due to polycationic PEI and not significantly different between the particle types (**Table S1**). Electron microscopy showed the solid particle core and a spherical particle shape (diameter of 72 ± 5 nm for CaP-P, 64 ± 5 nm for CaP-S and 64 ± 16 nm for CaP-S-C) (**Figure S1F**). Conjugation of nanoparticles with an anti-EGFR-IgG1-antibody, Cetuximab, did not significantly change the diameter of the particles. The difference in average particle diameter obtained between electron microscopy and DLS method is due to some particle aggregation in dispersion. Additionally, all preparations were tested for endotoxin presence which was determined to be below the maximum allowed level (determined <0,1 EU/ml, allowed 0,5 EU/ml) (25).

### Unsonicated Aggregated CaP-NPs Serve as Artificial Targets That Activate NK Cells

The aggregation state and colloidal stability are important parameters with respect to the biological applications of nanoparticles, but the effect of aggregated nanoparticles on NK cells has not been explored yet. We hypothesized that larger aggregates of nanoparticles could serve as artificial targets for NK cells and thus influence NK cell interaction with tumor cells. To test this hypothesis, after synthesis, lyophilized CaP-S and CaP-P NPs were resuspended in sterile distilled water and stored at 4°C for one to three weeks to allow for aggregation. The presence of the aggregates was confirmed by DLS measurement (**Figure 1A**). Thereafter, live cell imaging was performed on cocultures of NK cells, SKOV3 ovarian cancer cells and either freshly resuspended sonicated or long-term stored unsonicated CaP-NPs. From the recorded images (**Figure 1B**) and video (**Video S1A**, https://cloud.uk-essen.de/f/151ced3991ec41039707/?dl=1 and B, https://cloud.uk-essen.de/f/79fd353b893541918139/?dl=1) it can be seen that unsonicated nanoparticles formed aggregates (marked with black arrow; **Figure 1B**, right panel) that were of similar size as tumor cells. Functional NK degranulation assays revealed substantial NK cell degranulation when NK cells were coincubated with aggregated nanoparticles compared to the sonicated ones. (**Figure 1C**). Based on these data we further investigated effects of aggregated NPs in additional presence of ovarian cancer cells (**Figure 1D**). Addition of aggregated CaP-NPs to NK-tumor co-cultures still allowed the induction of substantial antibody-dependent cellular cytotoxicity (ADCC), which, in our experimental system, was induced by anti-EGFR antibody. However, the magnitude of ADCC was significantly reduced compared to control (no CaP-NP added) conditions, an effect that we did not observe for sonicated CaP-NPs. In summary, our data suggest, that aggregated CaP-NPs may cause unspecific and unintentional NK cell activation. This may finally hinder antitumoral NK cell activity against ovarian cancer cells under certain conditions, which could lead to reduced NK cell-mediated tumor cell lysis.

### Fresh and Sonicated Dispersed CaP-NPs Do Not Activate NK Cells

Based on these findings we turned to sonicated dispersed CaP-NPs and assessed the effects on NK viability and various parameters of NK activation. To prevent aggregation, CaP-NPs were sonicated for maximum 30 seconds, before addition to six hour coculture assays with NK cells. Viability assays showed that CaP-P nanoparticles were non-toxic, while CaP-S showed moderate toxicity when applied at higher doses (**Figure 2A**). This cytotoxicity of CaP-S coincided with a slight upregulation of the activation markers CD69 and NKG2D on the NK cell surface (**Figures 2B, C**). Expression of other surface markers such as CD16 (**Figure 2D**), DNAM-1 (data not shown) and NKp46 (data not shown) remained unchanged upon co-incubation with CaP-NPs. Using flow cytometry, IFNγ-staining (**Figure 2E**) and IFNγ-ELISpot (**Figure 2F**), it was confirmed that dispersed nanoparticles did not cause NK cell activation and any production or secretion of IFNγ compared to unstimulated controls. These results show that sonicated and dispersed CaP-NPs do not induce NK activation, except for minor induction of selected surface molecules in the presence of high NP doses.

### CaP-NPs Are Internalized by Ovarian Cancer Cells, Which Causes Cytotoxicity

After having analyzed effects of CaP-NPs on NK cells, we next investigated their interaction with ovarian cancer cells. To this end, we quantified the uptake of CaP-NPs, determined their cytotoxicity on cancer cells and monitored potential cell biological activation. Tumor cells were coincubated for 6h with nanoparticles before detachment and flow cytometry analysis. Results show that after the treatment the entire tumor cell population internalized CaP-NPs (**Figure S2A**) and fluorescence intensity was increasing with NP dose (**Figure 3A**). Additionally, Cetuximab conjugated silica nanoparticles (CaP-S-C) were taken up faster and stronger compared to CaP-S and CaP-P in OVCAR4 (**Figure 3A**). These results were additionally confirmed by microscopy pictures of coverslides seeded SKOV3 and OVCAR4 cells that were incubated for 3h with the CaP-NPs. There it can be seen that CaP-S-C are more dispersed compared to CaP-S, which appear as extracellular aggregates or on the cell surface (**Figure 3B**, indicated by red circles). Interestingly, CaP-P possess similar distribution as CaP-S-C. Viability of cancer cell lines OVCAR4 and SKOV3 was determined after 24 hour coincubation with various doses of sonicated CaP-P (**Figure 3C**), CaP-S (**Figure 3D**) and CaP-S-C (**Figure 3E**). Similar to **Figure 2** (cytotoxicity towards NK cells), only CaP-S showed toxicity against tumor cells.

Nanomaterials can cause immunomodulatory changes in the tumor microenvironment (TME). We tested whether CaP-NPs induced secretion of immunosuppressive cytokines or disbalance in tumor cell stress ligands, integrins or MHC class I complex, all important for susceptibility towards NK lysis. Supernatant of tumor cells treated with CaP-NPs was collected for measurement by ELISA and cells were analyzed by flow cytometry to determine expression of tumor cell stress ligand MIC-A (**Figure 4A**), ULBP-2,5,6 (**Figure 4B**), MHCI (**Figure 4C**) and CD54 (**Figure 4D**). In all cases, expression of markers remained unchanged, with a slight tendency of ULBP-2,5,6 downregulation in case of CaP-P addition. The quantification of secreted cytokines in the coculture supernatant after incubation of sonicated CaP-NPs showed a high degree of variability for IL-8 (**Figure 4E**). For both IL-8 and IL-6 (**Figure 4F**), nanoparticle addition did not induce strong or significant secretion changes when compared to vehicle control (H_2_O). These findings suggest that CaP-NPs do not significantly affect the expression of cytokines and ligands for NK receptors on ovarian cancer cells.

In order to address potential effects of aggregated nanomaterial on tumor cells we also determined the expression of stress ligands as well as tumor cell viability in the presence of aggregated CaP-NPs. As shown in **Figures S3A, B**, the expression levels of NK-activating ligands on tumor cells were lower in the presence of aggregated as compared to sonicated CaP-P NPs. Regarding tumor cell viability there was no difference of toxicity between aggregated and sonicated CaP-NPs (**Figure S3C**). In summary, our data suggest that in comparison to sonicated nanoparticles the aggregated nanomaterial may cause structural and functional changes on tumor cells but without affecting tumor cell viability.

### CaP-NPs Do Not Impair Ovarian Cancer Cell Lysis by NK Cells

In the final part of the study, we performed cocultures of OC and NK cells in the presence of CaP-NPs in order to test how the NPs influence the interaction of NKs with their tumor targets. To this end we analyzed: a) whether the CaP-NPs preferentially associate with OC or NK cells in co-cultures, b) whether the CaP-NPs induce changes in expression of functional surface markers on NK cells during the immune reaction, c) whether the CaP-NPs modulate natural and antibody-dependent cellular cytotoxicity (ADCC) of NKs against OC cells, d) whether Cetuximab that was coupled to CaP-NPs (CaP-S-C) retained ADCC-inducing activity. Our binding and uptake experiments show that all CaP-NP types are primarily taken up by tumor cells and not by the NKs. Only a portion of NK cells takes up or binds CaP-NPs (**Figure S2B**) and in case of CaP-S-C this is stronger than CaP-S or CaP-P (**Figure 5A**). The presence of CaP-NPs did not negatively affect conjugation between NK cells and SKOV3 cells, showing that contact to target cells is preserved (**Figure 5B**). We next analyzed effects of NPs on NK surface molecules under NK-tumor co-culture conditions. Especially in the presence of ADCC-inducing Cetuximab antibodies CaP-S modulated expression of NKG2D, CD69 and DNAM-1 (**Figures 5C–E**). No NP-induced changes were found for CD16 (data not shown) and NKp46 (data not shown).

**Figure 6:**
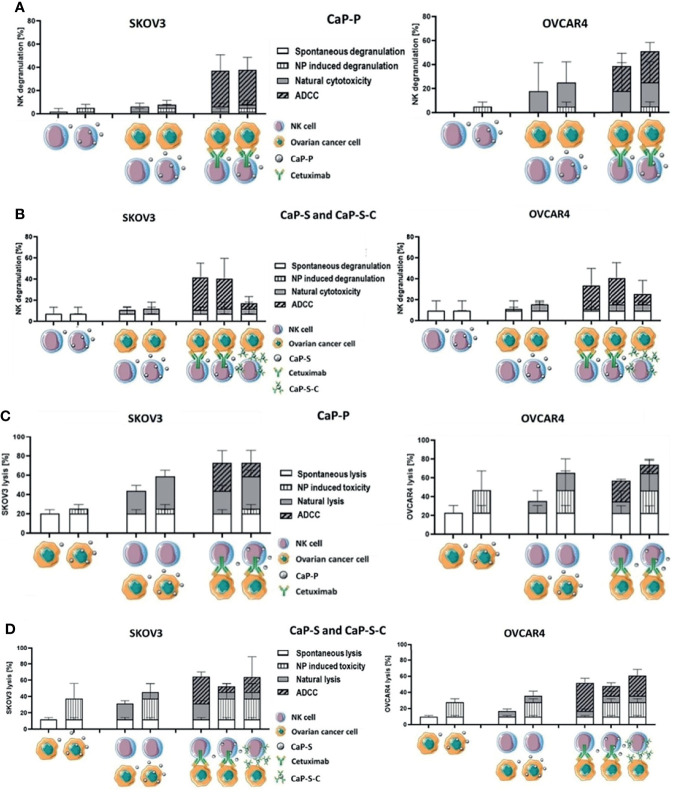
CaP-NPs do not inhibit NK cell reactivity and Cetuximab that is conjugated to NPs retains partial ADCC activity. Ovarian cancer cells, NK cells, Cetuximab and 12.5 µl of CaP-P **(A, C)** or CaP-S and CaP-S-C **(B, D)** were coincubated for 6 h or 24 h. Then **(A, B)** NK cell degranulation (CD107a assay) or **(C, D)** tumor cell lysis (Annexin/7AAD assay) were measured by flow cytometry. Cell schematics illustrate the experimental conditions. For each condition the spontaneous degranulation/lysis (background activity; 1st (starting from left) column), the NP-induced degranulation/toxicity (2nd column), natural cytotoxicity by NKs (3rd column) and ADCC (5th column) were determined and shown. Fourth column depicts natural cytotoxicity in the presence of NP and 6th column shows ADCC in the presence of NPs. The 7th column (only panels **B, D**) shows the degranulation/tumor lysis induced by Cetuximab that was coupled to NPs (CaP-S-C). A more direct comparison of the calculated NC and ADCC fractions of total degranulation (**Figure S4A**) or tumor lysis (**Figure S4B**) was included in the supplementary data. Both experiments were performed using OVCAR4 and SKOV3 cell lines. Three individual NK cell donors were used.

Lastly, we tested whether CaP-NPs would affect NK cell natural cytotoxicity and ADCC response, which is of the highest importance in our study. Since these responses are the main mechanisms by which NK cells exert antitumor activity it was of crucial relevance to investigate whether the CaP-NPs would impair these functions. To fully assess whether CaP-NPs cause any impairment on NK cell functionality we have calculated degranulation and tumor-killing contributions of individual components of the coculture system, using a formula explained in the materials and method section (Cetuximab, different CaP-NP species, NK cells). The detailed description of experimental conditions and calculated contributions is included in the legend to **Figure 6**. A more direct comparison of the calculated NC and ADCC fractions of total degranulation (**Figure S4A**) or tumor lysis (**Figure S4B**) was included in the supplementary data. The addition of dispersed CaP-P (**Figure 6A**) or CaP-S (**Figure 6B**) did not negatively affect NK natural degranulation or degranulation induced by soluble Cetuximab in both cocultures with SKOV3 or OVCAR4 as there was no significant difference in percentage of CD56+ CD107a+ NK cells between CaP-NP and control conditions. Similarly, the overall levels of tumor cell lysis under natural or antibody-mediated (ADCC) conditions were also maintained in the presence of CaP-P (**Figure 6C**). Lastly, it was tested whether CaP-NP coupled Cetuximab would retain ADCC-inducing functionality. To this end, the activity of CaP-S-C (Cetuximab coupled to CaP-S) was tested against its internal control CaP-S (**Figures 6B, D**). CaP-S-C conjugates retained NK degranulation inducing activity, although it was reduced compared to the soluble unconjugated cetuximab used in control conditions (**Figure 6B**). When total tumor lysis was assessed, it remained largely unchanged in the presence of CaP-S-C when compared to CaP-S (**Figure 6D**). It must be noted here that silica-coated NPs display some inherent toxicity for tumor cells (NP induced toxicity in **Figure 6D**) that seems to overlap with tumor cell killing induced by the Cetuximab antibody.

## Discussion

The medical nanotechnology has reached multiple medical fields, since the use of nanoparticles enables new options in diagnostics and offers novel possibilities in medical therapy. Because of their potential use as carriers for drugs and immunomodulators, nanoparticles also represent interesting tools for cancer immunotherapy (2). Among the broad variety of nanoparticles CaP-NPs have become attractive tools because of their biocompatibility, high mechanic stability and standardized producibility (4).

However, the functional activity of CaP-NPs within biological environments has not been explored sufficiently. Particularly, the impact on function of different components of the immune system is almost unknown. Thereby, it is mandatory for their use *in vivo* that nanocarriers do not impair immune cell activity. Single studies on osseous macrophages revealed that CaP-NPs enhanced inflammation by modulating macrophages while osteogenesis was attenuated (26). Other studies could show that CaP-NPs lead to maturation of antigen presenting cells, cytokine secretion and activation of cytotoxic T cells resulting in a strong immune response (9). Natural killer cells (NK cells) are among the first cells to interact with applied nanoparticles as these innate immune cells can act without prior sensitization. Previous studies demonstrated that cationic NPs augmented cytotoxic activity of NK cells while silver NPs enhanced expression of inhibitory receptors and thus reduced NK effector functions (27, 28). So far, no studies are available investigating the interaction between CaP-NPs and NK cells.

Therefore, in this study we investigated how CaP-NPs affect NK-tumor cell immune interactions. For this, we tested the effects of different preparations of CaP-NPs in an *in vitro* model of ovarian cancer immunotherapy. We cocultured NK cells and ovarian cancer cells in the presence of therapeutic anti-EGFR antibody Cetuximab and added triple-shell nanoparticles (CaP-P NPs), silica-shell nanoparticles (CaP-S NPs) as well as functionalized particles with Cetuximab bound to the surface of silica-shell-particles (CaP-S-C NPs). For visualization and uptake studies we included CaP-NPs with fluorescent dye. In our studies, we examined effects of nanoparticles on basic cell biological features of ovarian cancer cells. We mainly focused on the impact of CaP-NPs on cytotoxic and secretory functions of NK cells targeted against ovarian cancer cells and defined their role in interaction between NK and tumor cells.

First, we could observe that, over time, CaP-NPs tend to agglomerate in our culture system. It is known for some time that nanoparticles form aggregates when exposed to a biological environment (29). As interactions between CaP-NPs and NK cells have not been studied so far, we could show here for the first time that aggregates themselves may serve as artificial targets for NK cells, which may result in unspecific non-target cell directed cytotoxic NK cell activity. In line with this hypothesis, the magnitude of ADCC by NK in the presence of aggregated NP was reduced when compared to control conditions. In contrast, properly dispersed CaP-NP, which were generated by short sonication, did not lead to significant NK cell activation. These findings were supported by our data from DLS measuring particle size distribution. DLS data showed that sonication could completely prevent aggregation of CaP-P NPs, while some small aggregates (up to 1000 nm) were detected in sonicated CaP-S NPs. Furthermore, direct interaction between NK cells and aggregates could be observed during our live-cell imaging-studies. Here we could see that in coculture of NK and ovarian cancer cells aggregates of CaP-NPs substantially distracted NK cells from their original target. The potential biological relevance of this observation was demonstrated in our direct comparison of sonicated and unsonicated CaP-NPs influencing cytotoxic NK cell activity. Aggregated particles, especially CaP-S NPs, enhanced NK cell degranulation in contrast to sonicated CaP-NPs but in the presence of cocultured ovarian cancer cells the aggregated NPs tend deviate NK killing away from their intended cancer targets. Thus, we could show here, that standardized production and differentiated processing is mandatory before application of CaP-NPs *in vivo* in order to prevent adverse artificial immune cell activation and enable specific cellular modulation.

Further, we could demonstrate that dispersed CaP-NPs only showed slight immunotoxicity towards NK cells. This data is supported by other studies showing that CaP-NPs display low toxicity due to their composition of endogenous biomineral (4). However, certain degree of toxicity was observed in cocultures with CaP-S NPs. Furthermore, toxicity of CaP-S NPs was correlated to enhanced NK cell degranulation and to a tendential upregulation of CD69. The advantage of silica shell-containing CaP-S NPs is the possibility of their functionalization since proteins or antibodies cannot be covalently bound onto the calcium phosphate surface (8). Due to our results, certain components which are prerequisites for functionalization or contribute to the stabilization of particles can lead to a slight increase in toxicity but do not lead to substantial impairment of NK function.

In further studies we wanted to define the role of CaP-NPs during the interaction between NK and ovarian cancer cells. Principally, we could demonstrate that cytotoxic and secretory function of NK cells and NK-mediated tumor cell lysis remained unaffected by different preparations of CaP-NPs. In parallel, we investigated the expression of different NK cell markers. We could observe that natural cytotoxicity as well as antibody-dependent cellular cytotoxicity (ADCC) were accompanied by an upregulation of NKG2D and CD69 and by downregulation of DNAM-1 which indicates NK cell activation. Interestingly, Cetuximab-functionalized CaP-S-C NPs were still able to induce ADCC and tumor cell lysis. However, the antibody conjugation onto the nanoparticle surface lead to reduced Cetuximab activity (30). One possible explanation could be that conjugation of randomly orientated antibodies leads to geometrical inaccessibility or inactivation of Fc and Fab antibody fragments which could be responsible for reduced ADCC (31). Another possible mechanism could be, that nanoparticle-bound Cetuximab is taken up faster by tumor cells and therefore inaccessible for NK cell interaction in contrast to free Cetuximab. Even though CaP-S-C nanoparticles were noted to induce lower NK degranulation in our study, the degree of tumor lysis is comparable to the ADCC induced by free Cetuximab which could be due to a fundamentally slightly increased cytotoxicity of CaP-S-C. Related to NK tumor cell interaction we also investigated formation of conjugates in presence of different types of CaP-NPs. Most importantly, addition of CaP-NPs did not impair the conjugate formation being mandatory for tumor immune cell interaction. In conclusion, CaP-NPs did not interfere with antitumoral NK activity but rather induced a slight activation.

In further experiments we studied the uptake of CaP-NPs by ovarian cancer cells. We could show that all types of CaP-NPs were taken up by tumor cells in a dose-dependent manner. In detail, microscopic as well as flow cytometry proved that functionalized CaP-S-C NP and to a lesser extent also CaP-P NPs displayed the highest uptake rate. In contrast, CaP-S NPs tend to form bigger aggregates (up to 10 µm diameter) which may explain their lower uptake rate and localization on the surface of the tumor cell. In a coculture of tumor cell and NK cells all types of CaP-NPs were predominantly bound to or taken up by ovarian cancer cells while only a small faction interacted with NK cells. Interestingly, NK cells bind CaP-S-C stronger than CaP-S NPs which could indicate antibody recognition by NK cells. Basically, in biological fluids cellular uptake of CaP-NPs is dependent on size, charge and surface characteristics (8, 32). So, different components like PEI, silica shell or specific antibodies determine state of aggregation, absorption of proteins and cellular uptake (33–35). An alternative explanation could be the targeting-effect by Cetuximab in CaP-S-C NPs, which has been already described in principle (36). In summary, these findings show that CaP-NPs do not impair the tumor cytolytic activity of NK cells and may serve as carriers for therapeutic functional modulation of tumor cell targets.

Interestingly, the intracellular uptake of CaP-NPs in ovarian cancer cells did not lead to altered expression of stress-induced ligands (MIC A and ULBP 2, 5, 6). Additionally, MHC-I and CD54, crucial receptors for NK cell interaction, also remained unaffected. This suggests that recognition by immune cells would be continuously enabled (37). This is consistent with our observation that uptake of CaP-P and CaP-S-C did not cause any direct toxicity. This was not to be expected, especially since the lysosomal escape caused by PEI may lead to necrosis in high particle concentration (38, 39). According to this, we did also not observe any enhanced secretion of tumor cytokines (IL-6 and IL-8) which can also serve as measure for toxicity of nanoparticles (40). In contrast, CaP-S NPs induced moderate dose-dependent direct toxicity which would argue for a toxic effect of the silica shell. Summarizing our data, with the exception of silica-shell particles, CaP-NPs do not exert direct, unspecific cytotoxicity, which would qualify them for a differentiated immunotherapeutic approach.

In this study we directly added CaP-NPs to tumor-NK cocultures and assessed their direct potential effects on NK and tumor cells as well as on their interaction between tumor and NK cells. In prelimininary work to this project, we also investigated whether a pretreatment of tumor cells with different preparations of CaP-NPs could affect susceptibility of OC cells towards NK cell cytotoxicity. To this end, we pre-incubated tumor cells with the different CaP-NPs and measured natural cytotoxicity and ADCC of added NK cells as well as tumor cell lysis. However, no differences between both settings were observed (data not shown) further supporting the idea that CaP-NPs do not impair antitumor activity of NK cells.

In summary, in this study we could demonstrate that CaP-NPs slightly activate NK cells and do not impair cytotoxic and secretory NK functions. Importantly, aggregated nanoparticles which were not dispersed by sonication may serve as artificial target and deviate NK cells away from their intended targets. Furthermore, Cetuximab-functionalized CaP-NPs preserved ADCC functionality. In conjunction, by confirming CaP-NP biocompatibility, low toxicity and demonstrating efficient uptake in ovarian cancer cells our study offers options for integration of CaP-NPs in prospective immunotherapeutic concepts for cancer therapy.

## Data Availability Statement

The original contributions presented in the study are included in the article/**Supplementary Material**. Further inquiries can be directed to the corresponding author.

## Ethics Statement

The studies involving human participants were reviewed and approved by Ethikkommission der Medizinischen Fakultät der Universität Duisburg-Essen. The participants provided their written informed consent to participate in this study.

## Author Contributions

AH, SBr, MO, and NM-G conceived and planned the experiments. AH, MS, MO, SBe, and SK carried out the experiments. AH, MS, MO, SBe, SBr, and NM-G analyzed and interpreted the data. PAH, SK, and ME provided crucial materials and reagents. AH, NM-G, and SBr wrote the manuscript. MS, ME, and SK edited the manuscript. SBr and NM-G supervised the project. All authors reviewed or contributed to the final manuscript.

## Funding

This work was supported by the Deutsche Forschungsgemeinschaft (DFG, German Research Foundation) which was awarded to NM-G and SBr (project numbers: MA 7926/2-1, BR 2278/5-1).

## Conflict of Interest

The authors declare that the research was conducted in the absence of any commercial or financial relationships that could be construed as a potential conflict of interest.

## Publisher’s Note

All claims expressed in this article are solely those of the authors and do not necessarily represent those of their affiliated organizations, or those of the publisher, the editors and the reviewers. Any product that may be evaluated in this article, or claim that may be made by its manufacturer, is not guaranteed or endorsed by the publisher.

## Abbreviations

NC, natural cytotoxicity; NK, NK cells; TC, tumor cells; NP, NP induced degranulation; ADCC, antibody dependent cell-mediated cytotoxicity.

## References

[1] Rathor S Bhatt DC Aamir S Singh SK Kumar VA. A Comprehensive Review on Role of Nanoparticles in Therapeutic Delivery of Medicine. Pharm Nanotechnol (2017) 5(4):263–75. doi: 10.2174/2211738505666171113130639 29141578

[2] Alsaab HO Al-Hibs AS Alzhrani R Alrabighi KK Alqathama A Alwithenani A. Nanomaterials for Antiangiogenic Therapies for Cancer: A Promising Tool for Personalized Medicine. Int J Mol Sci (2021) 22(4):1631. doi: 10.3390/ijms22041631 33562829 PMC7915670

[3] Ewen ST Fauzi A Tang YQ Chamyuang S Chia A. A Review on Advances of Treatment Modalities for Alzheimer's Disease. Life Sci (2021) 276:119129. doi: 10.1016/j.lfs.2021.119129 33515559

[4] Epple M. Review of Potential Health Risks Associated With Nanoscopic Calcium Phosphate. Acta Biomater (2018) 77:1–14. doi: 10.1016/j.actbio.2018.07.036 30031162

[5] Tadic D Epple M. A Thorough Physicochemical Characterisation of 14 Calcium Phosphate-Based Bone Substitution Materials in Comparison to Natural Bone. Biomaterials (2004) 25(6):987–94. doi: 10.1016/S0142-9612(03)00621-5 14615163

[6] Levingstone TJ Herbaj S Dunne NJ. Calcium Phosphate Nanoparticles for Therapeutic Applications in Bone Regeneration. Nanomater (Basel) (2019) 9(11):1570. doi: 10.3390/nano9111570 PMC6915504 31698700

[7] Enax J Epple M. Synthetic Hydroxyapatite as a Biomimetic Oral Care Agent. Oral Health Prev Dent (2018) 16(1):7–19. doi: 10.3290/j.ohpd.a39690 29335686

[8] Sokolova V Epple M. Biological and Medical Applications of Calcium Phosphate Nanoparticles. Chemistry (2021) 27:7471–88. doi: 10.1002/chem.202182761 PMC8251768 33577710

[9] Scheffel F Knuschke T Otto L Kollenda S Sokolova V Cosmovici C. Effective Activation of Human Antigen-Presenting Cells and Cytotoxic CD8(+) T Cells by a Calcium Phosphate-Based Nanoparticle Vaccine Delivery System. Vaccines (Basel) (2020) 8(1):110. doi: 10.3390/vaccines8010110 PMC7157756 32121590

[10] Knuschke T Rotan O Bayer W Kollenda S Dickow J Sutter K. Induction of Type I Interferons by Therapeutic Nanoparticle-Based Vaccination Is Indispensable to Reinforce Cytotoxic CD8(+) T Cell Responses During Chronic Retroviral Infection. Front Immunol (2018) 9:614. doi: 10.3389/fimmu.2018.00614 29740425 PMC5924795

[11] Tenkumo T Vanegas Sáenz JR Takada Y Takahashi M Rotan O Sokolova V. Gene Transfection of Human Mesenchymal Stem Cells With a Nano-Hydroxyapatite-Collagen Scaffold Containing DNA-Functionalized Calcium Phosphate Nanoparticles. Genes Cells (2016) 21(7):682–95. doi: 10.1111/gtc.12374 27238217

[12] Chernousova S Epple M. Live-Cell Imaging to Compare the Transfection and Gene Silencing Efficiency of Calcium Phosphate Nanoparticles and a Liposomal Transfection Agent. Gene Ther (2017) 24(5):282–9. doi: 10.1038/gt.2017.13 PMC5442419 28218744

[13] Pittella F Miyata K Maeda Y Suma T Watanabe S Chen Q. Pancreatic Cancer Therapy by Systemic Administration of VEGF siRNA Contained in Calcium Phosphate/Charge-Conversional Polymer Hybrid Nanoparticles. J Control Release (2012) 161(3):868–74. doi: 10.1016/j.jconrel.2012.05.005 22580114

[14] Tobin LA Xie Y Tsokos M Chung SI Merz AA Arnold MA. Pegylated siRNA-Loaded Calcium Phosphate Nanoparticle-Driven Amplification of Cancer Cell Internalization *In Vivo* . Biomaterials (2013) 34(12):2980–90. doi: 10.1016/j.biomaterials.2013.01.046 PMC3633203 23369215

[15] Zhang N Lu C Shu G Li J Chen M Chen C. Gadolinium-Loaded Calcium Phosphate Nanoparticles for Magnetic Resonance Imaging of Orthotopic Hepatocarcinoma and Primary Hepatocellular Carcinoma. Biomater Sci (2020) 8(7):1961–72. doi: 10.1039/C9BM01544B 32064471

[16] Shetab Boushehri MA Lamprecht A. Nanoparticles as Drug Carriers: Current Issues With *In Vitro* Testing. Nanomed (Lond) (2015) 10(21):3213–30. doi: 10.2217/nnm.15.154 26548350

[17] Hannon G Lysaght J Liptrott NJ Prina-Mello A. Immunotoxicity Considerations for Next Generation Cancer Nanomedicines. Adv Sci (Weinh) (2019) 6(19):1900133. doi: 10.1002/advs.201900133 31592123 PMC6774033

[18] Caracciolo G Farokhzad OC Mahmoudi M. Biological Identity of Nanoparticles *In Vivo*: Clinical Implications of the Protein Corona. Trends Biotechnol (2017) 35(3):257–64. doi: 10.1016/j.tibtech.2016.08.011 27663778

[19] Moretta L Pietra G Montaldo E Vacca P Pende D Falco M. Human NK Cells: From Surface Receptors to the Therapy of Leukemias and Solid Tumors. Front Immunol (2014) 5:87. doi: 10.3389/fimmu.2014.00087 24639677 PMC3945935

[20] Screpanti V Wallin RPA Grandien A Ljunggren HG. Impact of FASL-Induced Apoptosis in the Elimination of Tumor Cells by NK Cells. Mol Immunol (2005) 42(4):495–9. doi: 10.1016/j.molimm.2004.07.033 15607805

[21] Alter G Malenfant JM Altfeld M. CD107a as a Functional Marker for the Identification of Natural Killer Cell Activity. J Immunol Methods (2004) 294(1-2):15–22. doi: 10.1016/j.jim.2004.08.008 15604012

[22] Kollenda SA Klose J Knuschke T Sokolova V Schmitz J Staniszewska M. In Vivo Biodistribution of Calcium Phosphate Nanoparticles After Intravascular, Intramuscular, Intratumoral, and Soft Tissue Administration in Mice Investigated by Small Animal PET/CT. Acta Biomater (2020) 109:244–53. doi: 10.1016/j.actbio.2020.03.031 32251787

[23] Kozlova D Chernousova S Knuschke T Buer J Westendorf AM. Cell Targeting by Antibody-Functionalized Calcium Phosphate Nanoparticles. J Mater Chem (2012) 22:396–404. doi: 10.1039/C1JM14683A

[24] Burshtyn DN Davidson C. Natural Killer Cell Conjugate Assay Using Two-Color Flow Cytometry. Methods Mol Biol (2010) 612:89–96. doi: 10.1007/978-1-60761-362-6_7 20033636

[25] US Department of Health and Human Services/Public Health Services/Food and Drug Administration. Guideline on Validation of the Limulus Amebocyte Lysate Test as an End-Product Endotoxin Test for Human and Animal Parental Drugs, Biological Products and Medical Devices (1987). Available at: http://www.fda.gov/cber/gdlns/lal.pdf.

[26] Chen L Qiao P Liu H Shao L. Amorphous Calcium Phosphate NPs Mediate the Macrophage Response and Modulate BMSC Osteogenesis. Inflammation (2021) 44(1):278–96. doi: 10.1007/s10753-020-01331-9 32939669

[27] Kim KS Han JH Choi SH Jung HY Park JD An HJ. Cationic Nanoparticle-Mediated Activation of Natural Killer Cells for Effective Cancer Immunotherapy. ACS Appl Mater Interfaces (2020) 12(51):56731–40. doi: 10.1021/acsami.0c16357 33290037

[28] Müller L Steiner SK Rodriguez-Lorenzo L Petri-Fink A Rothen-Rutishauser B Latzin P. Exposure to Silver Nanoparticles Affects Viability and Function of Natural Killer Cells, Mostly *via* the Release of Ions. Cell Biol Toxicol (2018) 34:167–76. doi: 10.1007/s10565-017-9403-z 28721573

[29] Rausch K Reuter A Fischer K Schmidt M. Evaluation of Nanoparticle Aggregation in Human Blood Serum. Biomacromolecules (2010) 11(11):2836–9. doi: 10.1021/bm100971q 20961117

[30] Ahmed M Pan DW Davis ME. Lack of *In Vivo* Antibody Dependent Cellular Cytotoxicity With Antibody Containing Gold Nanoparticles. Bioconjug Chem (2015) 26(5):812–6. doi: 10.1021/acs.bioconjchem.5b00139 PMC4445771 25879583

[31] Iijima M Araki K Liu Q Somiya M Kuroda S. Oriented Immobilization to Nanoparticles Enhanced the Therapeutic Efficacy of Antibody Drugs. Acta Biomater (2019) 86:373–80. doi: 10.1016/j.actbio.2019.01.011 30641288

[32] Walkey CD Olsen JB Guo H Emili A Chan WC. Nanoparticle Size and Surface Chemistry Determine Serum Protein Adsorption and Macrophage Uptake. J Am Chem Soc (2012) 134(4):2139–47. doi: 10.1021/ja2084338 22191645

[33] Xia T Kovochich M Liong M Meng H Kabehie S George S. Polyethyleneimine Coating Enhances the Cellular Uptake of Mesoporous Silica Nanoparticles and Allows Safe Delivery of siRNA and DNA Constructs. ACS Nano (2009) 3(10):3273–86. doi: 10.1021/nn900918w PMC3900639 19739605

[34] Wartlick H Michaelis K Balthasar S Strebhardt K Kreuter J Langer K. Highly Specific HER2-Mediated Cellular Uptake of Antibody-Modified Nanoparticles in Tumour Cells. J Drug Target (2004) 12(7):461–71. doi: 10.1080/10611860400010697 15621671

[35] Wen L Wang Q Zheng T Chen J. Effects of Polyethylenimine on the Dispersibility of Hollow Silica Nanoparticles. Front Chem Eng China (2007) 1:277–82. doi: 10.1007/s11705-007-0050-4

[36] Tseng SH Chou MY Chu IM. Cetuximab-Conjugated Iron Oxide Nanoparticles for Cancer Imaging and Therapy. Int J Nanomed (2015) 20(10):3663–85. doi: 10.2147/IJN.S80134 PMC4445874 26056447

[37] Barber DF Faure M Long EO. LFA-1 Contributes an Early Signal for NK Cell Cytotoxicity. J Immunol (2004) 173(6):3653–9. doi: 10.4049/jimmunol.173.6.3653 15356110

[38] Akinc A Thomas M Klibanov AM Langer R. Exploring Polyethylenimine-Mediated DNA Transfection and the Proton Sponge Hypothesis. J Gene Med (2005) 7:657–63. doi: 10.1002/jgm.696 15543529

[39] Liu Z Xiao Y Chen W Wang Y Wang B Wang G. Calcium Phosphate Nanoparticles Primarily Induce Cell Necrosis Through Lysosomal Rupture: The Origination of Material Cytotoxicity. J Mater Chem B (2014) 2(22):3480–9. doi: 10.1039/c4tb00056k 32261468

[40] Elsabahy M Wooley KL. Cytokines as Biomarkers of Nanoparticle Immunotoxicity. Chem Soc Rev (2013) 42(12):5552–76. doi: 10.1039/c3cs60064e PMC3665642 23549679

