# Supplementary material for: Reactivity of NK Cells Against Ovarian Cancer Cells Is Maintained in the Presence of Calcium Phosphate Nanoparticles
Source: Front Immunol. 2022 Feb 18;13:830938. doi: 10.3389/fimmu.2022.830938 (PMC8895254; doi:10.3389/fimmu.2022.830938)
Supplement: Supplementary file 1 [file DataSheet_1.pdf]

frontiers

Supplementary Material

Nanoparticle type	DLS [nm]	SEM [nm]	PDI	Zeta Potential [mV]	[Ca2+] [µg/mL]	CaP Conc. [µg/mL]	Particles/mL	Antibodies [mg/mL]
**CaP-PEI -CaP-PEI**	**129 ± 32**	**57 ± 9**	**0.343**	**18.0 ± 1.4**	**62.50**	**156.88**	**$5.12 \cdot 10^{11}$**	**-**
**CaP-PEI_FITC-CaP-PEI**	**131 ± 19**	**72 ± 5**	**0.262**	**17.9 ± 0.8**	**39.70**	**99.65**	**$1.61 \cdot 10^{11}$**	**-**
**CaP-PEI -SiO_2-SH**	**309 ± 49**	**58 ± 7**	**0.140**	**22.2 ± 0.4**	**46.55**	**116.84**	**$3.62 \cdot 10^{11}$**	**-**
**CaP-PEI_FITC-SiO_2-SH**	**122 ± 32**	**64 ± 5**	**0.382**	**28.2 ± 1.2**	**62.50**	**156.88**	**$3.62 \cdot 10^{11}$**	**-**
**CaP-PEI -SiO_2-S-Cetuximab**	**97 ± 19**	**88 ± 15**	**0.151**	**18.8 ± 1.5**	**9.30**	**23.34**	**$2.07 \cdot 10^{10}$**	**0.058**
**CaP-PEI_FITC-SiO_2-S-Cetuximab**	**123 ± 26**	**64 ± 16**	**0.246**	**23.8 ± 2.7**	**34.83**	**87.42**	**$2.02 \cdot 10^{11}$**	**0.053**

Supplementary Table S1. Representative physicochemical properties of different CaP-NPs. Notice that different NP species have comparatively similar properties such as size, potential, and particle number.

Supplementary Figure S1.: Calcium phosphate nanoparticles are biocompatible state of art produced nanoparticles. (A) Schematic representations of the chemical steps needed for the synthesis of different calcium phosphate nanoparticles. **(B)** CaP/PEI/SiO_2-SH – Silica coated CaP-NPs (referred as **CaP-S**), **(C)** CaP/PEI/SiO_2-S-CET – Silica coated CaP-NPs functionalized with Cetuximab antibody (referred as **CaP-S-C**), **(D)** CaP/PEI/CaP/PEI – Triple shell CaP-NPs (referred as **CaP-P**). **(E)** Representative nanoparticle size and distribution quantified by dynamic light scattering. **(F)** Representative nanoparticle micrographs obtained by scanning electron microscopy.

Supplementary Figure S2.: Uptake of CaP-NPs in cancer cell mono-cultures and co-cultures with NK cells. (A) Representative plot showing the uptake of CaP-NPs by SKOV3 cancer cells from Fig. 4.A after 6 hours, analyzed by flow cytometry. **(B)** Representative plot showing the uptake of 12 µl of CaP-NPs in the NK-TC coculture after 3 hours from Fig 6.A, analyzed by flow cytometry.

Supplementary Figure S3.: Effect of aggregated and sonicated CaP-NPs on cancer cell stress marker expression and tumor cell viability. SKOV3 OC cells were coincubated with 12,5 µl of sonicated or aggregated CaP-P and CaP-S nanoparticle suspensions for 24 h and expression of MICA **(A)** and ULBP-2/5/6 **(B)** was determined by surface staining flow cytometry. **(C)** SKOV3 cell lysis after 24 h coincubation with aggregated or sonicated CaP-NPs was determined by Annexin/7AAD flow cytometry staining. Data points represent three individual experiments. Significance testing was done using paired t-test, significance is assumed for $p<0,1$ (*), $<0,01$ (**). No significance between compared groups indicated by ns.

Supplementary Figure S4.: Simplified comparison of the calculated NC and ADCC. NK degranulation **(A)** and tumor lysis **(B)** in control conditions and the presence of CaP-NPs using data shown in the Fig 6. Both experiments were performed using SKOV3 (left panels) and OVCAR4 (right panels) cell lines. Each data point represents an individual NK cell donor, N=3. Significance testing was done using both unpaired t-test with Welch's correction and one-way ANOVA with posthoc Dunnets multiple comparison test, significance is assumed for $p < 0,05$ (*), $<0,01$ (**). No significance between compared groups indicated by ns.

Supplementary Figure S5.: Representative flow cytometry gating strategy for **(A)** NK Degranulation, **(B)** Tumor killing, **(C)** NK-Tumor cell conjugate assay and **(D)** Tumor cell stress ligand staining.

Supplementary Figure S6.: Graphical abstract and summary of the most important findings in the study. Figure was assembled using open-source illustrations provided by Sevier Medical Art (smart.servier.com).

Supplementary Video S1.: Fluorescent CaP-NPs (red) interact differently with NK cells depending if they are **(A)** aggregated or fresh and **(B)** sonicated. Notice how NK cells are interacting with NP aggregates compared to the sonicated dispersed NP preparation. Tumor cells marked in green. Magnification 20x. Intervals 4 minutes. Duration 3,8h.
