# Supplementary figures and images for: Reactivity of NK Cells Against Ovarian Cancer Cells Is Maintained in the Presence of Calcium Phosphate Nanoparticles
Source: Front Immunol. 2022 Feb 18;13:830938. doi: 10.3389/fimmu.2022.830938 (PMC8895254; doi:10.3389/fimmu.2022.830938)

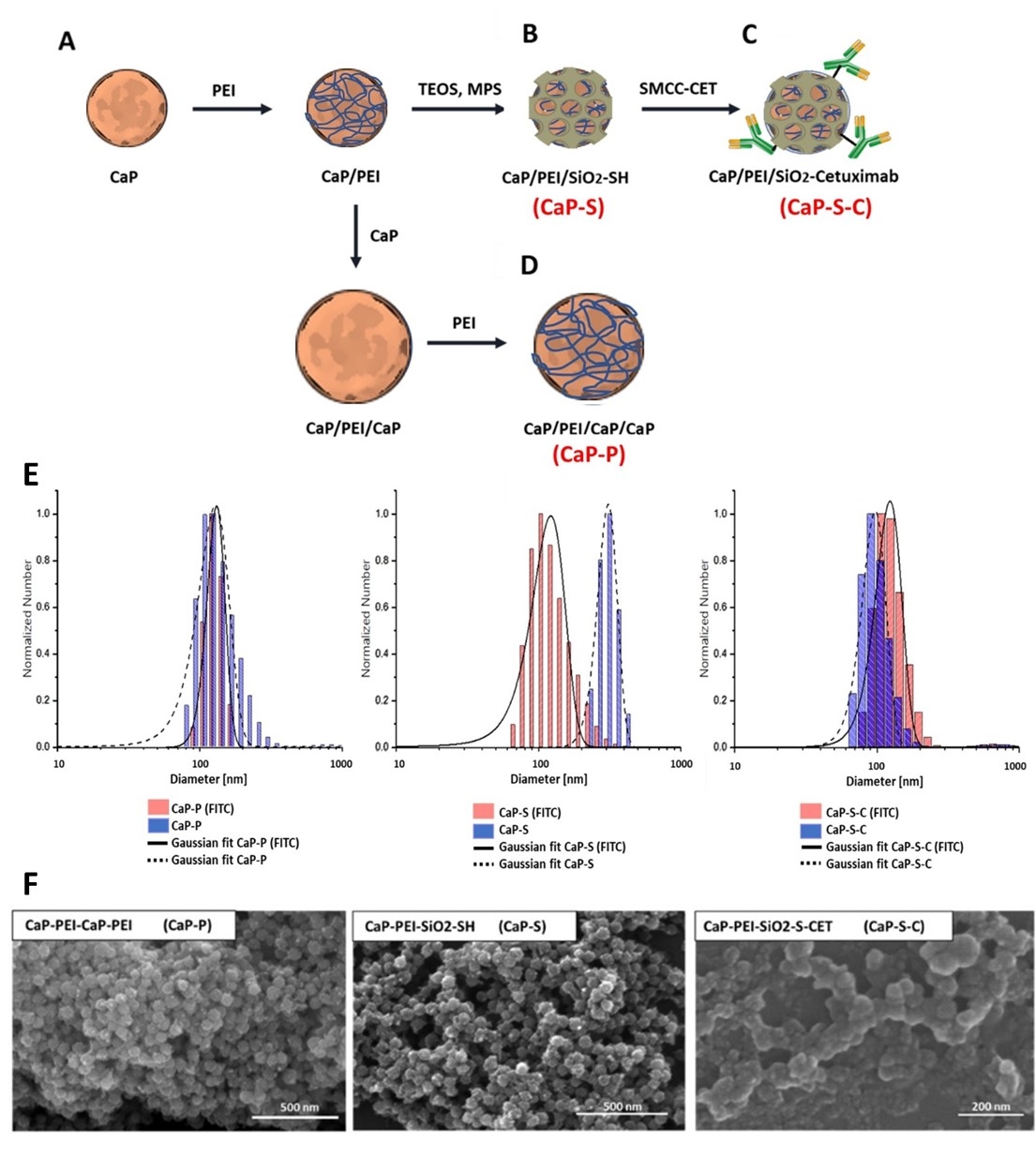

Supplement: Supplementary file 2 [file Image_1.jpeg]

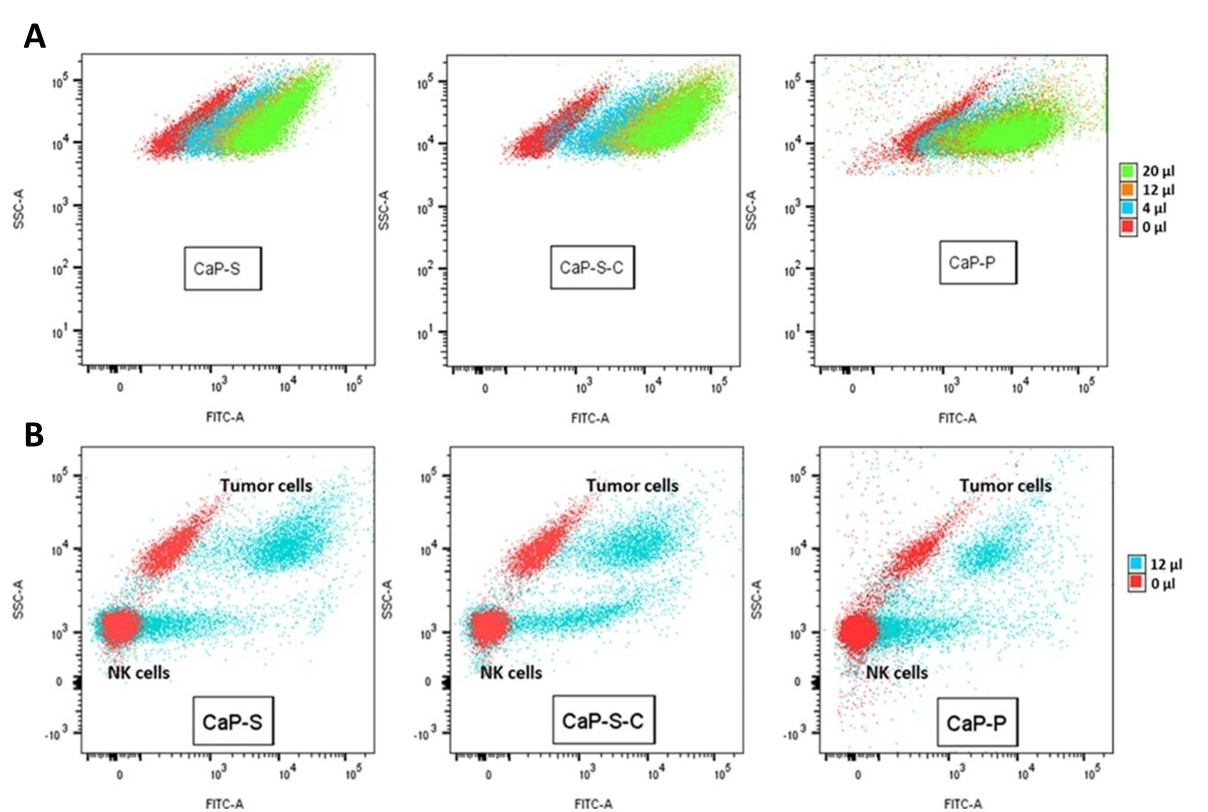

Supplement: Supplementary file 3 [file Image_2.jpeg]

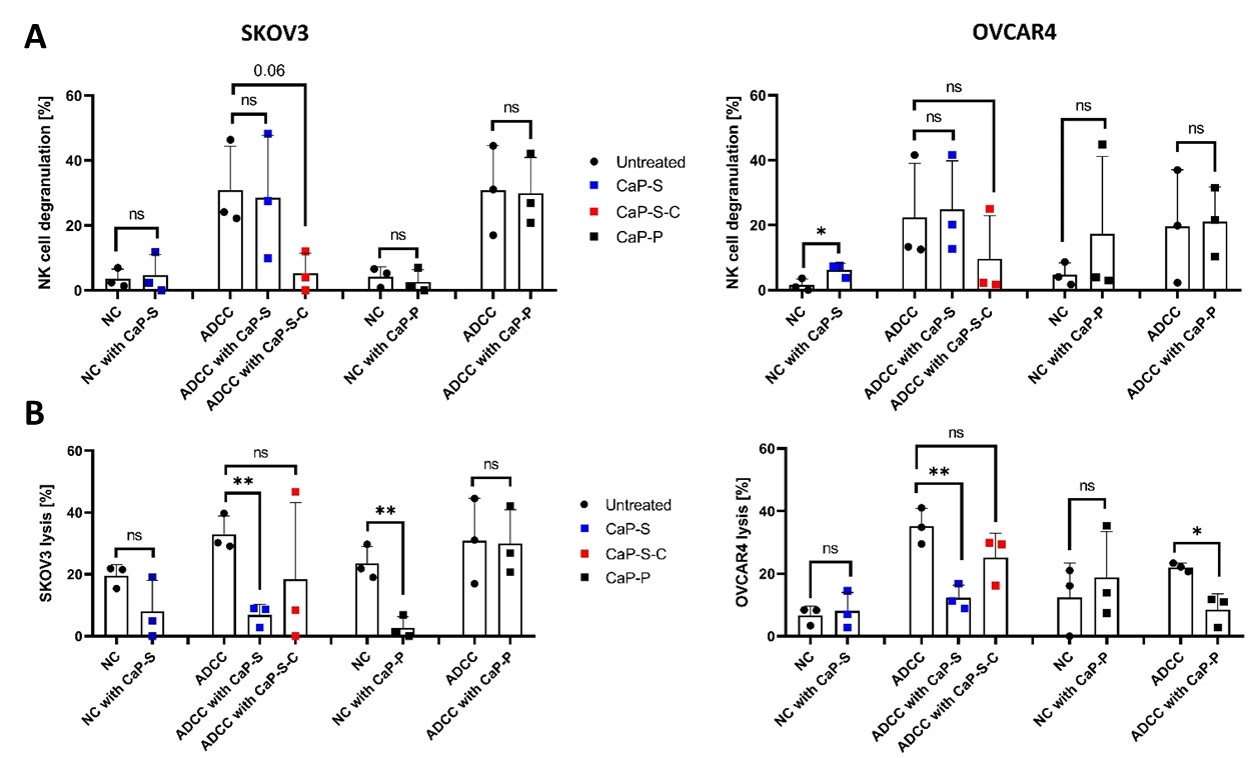

Supplement: Supplementary file 5 [file Image_4.jpeg]

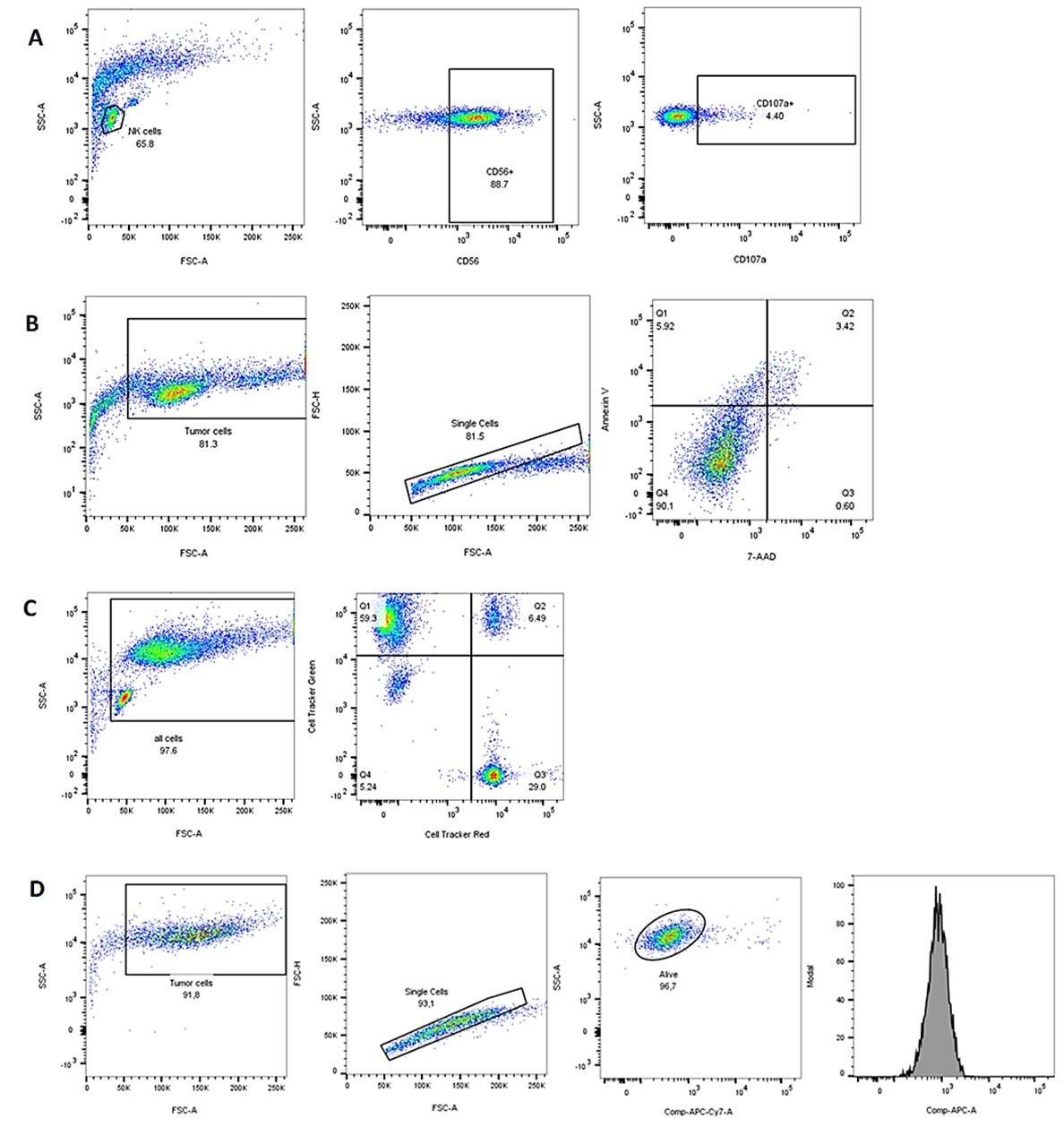

Supplement: Supplementary file 6 [file Image_5.jpeg]

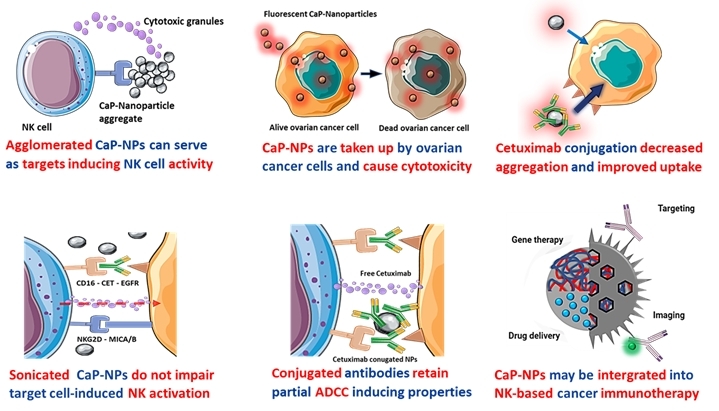

Supplement: Supplementary file 7 [file Image_6.jpeg]
